# Global emergence of Enterovirus 71: a systematic review

**DOI:** 10.1186/s43088-022-00258-4

**Published:** 2022-06-13

**Authors:** Gayatree Nayak, Sanat Kumar Bhuyan, Ruchi Bhuyan, Akankshya Sahu, Dattatreya Kar, Ananya Kuanar

**Affiliations:** 1 Centre for Biotechnology, Siksha O Anusandhan (Deemed to Be) University, Kalinga Nagar, Ghatikia, Bhubaneswar, Odisha 751003 India; 2 Institute of Dental Sciences, Siksha ‘O’ Anusandhan (Deemed to Be) University, Bhubaneswar, Odisha 751003 India; 3 Department of Medical Research, Health Science, IMS and SUM Hospital, Siksha O Anusandhan (Deemed to Be) University, Bhubaneswar, Odisha 751003 India

**Keywords:** Enterovirus 71, HFMD, Neurological disorders, Molecular epidemiology, Environmental factors, Vaccine, Children

## Abstract

**Background:**

Hand, foot, and mouth disease (HFMD) is a viral infection caused by a virus from the enterovirus genus of picornavirus family that majorly affects children. Though most cases of HFMD do not cause major problems, the outbreaks of Enterovirus 71 (EV71) can produce a high risk of neurological sequelae, including meningoencephalitis, lung difficulties, and mortality. In Asia, HFMD caused by EV71 has emerged as an acutely infectious disease of highly pathogenic potential, which demands the attention of the international medical community.

**Main body of the abstract:**

Some online databases including NCBI, PubMed, Google Scholar, ProQuest, Scopus, and EBSCO were also accessed using keywords relating to the topic for data mining. The paid articles were accessed through the Centre Library facility of Siksha O Anusandhan University. This work describes the structure, outbreak, molecular epidemiology of Enterovirus 71 along with different EV71 vaccines. Many vaccines have been developed such as inactivated whole-virus live attenuated, subviral particles, and DNA vaccines to cure the patients. In Asia–Pacific nations, inactivated EV71 vaccination still confronts considerable obstacles in terms of vaccine standardization, registration, price, and harmonization of pathogen surveillance and measurements.

**Short conclusion:**

HFMD has emerged as a severe health hazard in Asia–Pacific countries in recent decades. In Mainland China and other countries with high HFMD prevalence, the inactivated EV71 vaccination will be a vital tool in safeguarding children's health. When creating inactivated EV71 vaccines, Mainland China ensured maintaining high standards of vaccine quality. The Phase III clinical studies were used to confirm the safety and effectiveness of vaccinations.

**Graphical Abstract:**

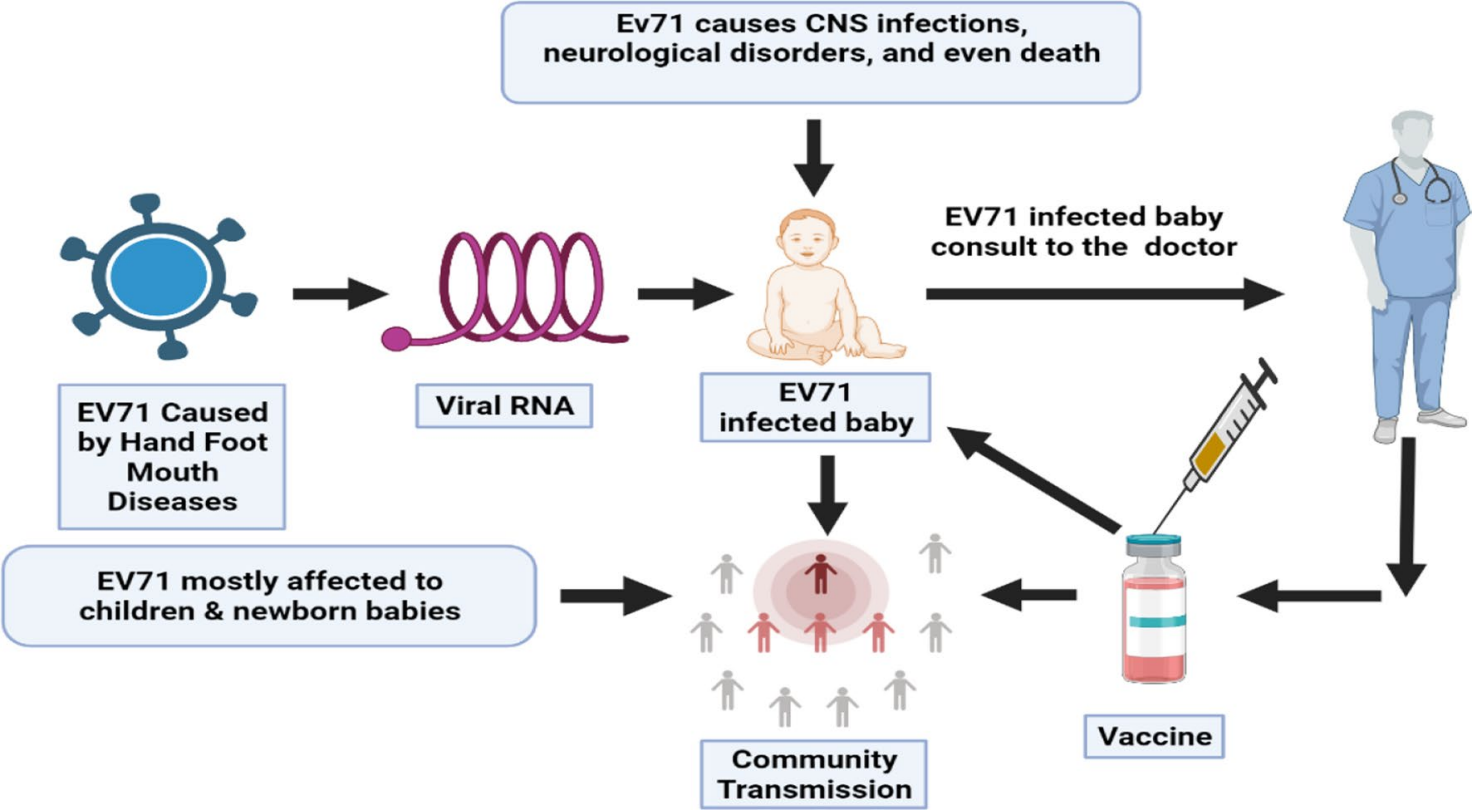

## Background

The EV71 virus generally causes hand, foot, and mouth diseases (HFMDs) or herpangina and neurological abnormalities among children below 5 to7 years old. It causes severe rashes in the hand, foot, and mouth that appear like blisters [1]. The symptoms also include fever and painful blister-like sores. This virus can transmit directly from an infected person to another through faecal materials, saliva, sputum, respiratory droplets, etc. [2, 3]. Moreover, EV71 can also transmit indirectly through steel, iron, paper, plastic, and other things that come in contact with infected persons [4]. Generally, children between the age group of 5 and 7 years get easily affected by the EV71 virus due to their low immunity, whereas adults rarely get affected by this virus as they are mostly immunogenic.

HFMD causes severe health hazards such as abnormality in CNS, respiratory problems, cardiovascular disorders, aseptic meningitis, cerebella ataxia, poliomyelitis-like paralysis acute brainstem encephalitis, cardiopulmonary failure, fulminant neurogenic pulmonary oedema, and also increase in death rates [5]. HFMD is mostly triggered in environmental conditions of high temperature, warm regions, or mostly in summer and spring seasons 2 and 5 and also spreads fast in warm or humid areas [6].

HFMD can occur more by EV71 but less by coxsackieviruses as A16 (CVA16), A5, A6, A7, A9, A10, B1, B2, B3, and B5 [7, 8]. Enterovirus 71 is also called as Enterovirus A71 (EV-A71), belonging to the family Picornaviridae. Sometimes, EV71 causes polio-like disorder in children and encephalitis [9]. According to Chen et al. [10], EV-A71 infections pose a higher risk to boys than girls, but still the mechanism of action of this virus is not clear. EV71 infection is a major threat to public health all over the world [10, 11].

Solomon et al. [4] reported that more than 100 human enterovirus serotypes are present including 3 polioviruses, 23 coxsackieviruses A (CA), 6 coxsackieviruses B (CB), 31 echoviruses, and 39 numbered enteroviruses (EV68-71, EV73-102, EV104-107, and EV109). The infections are mostly detected in children and very rarely in adults. Further, Lee et al. [12] identified that human enteroviruses are phylogenetically divided into 4 types: A, B, C, and D. However, these different strains of EV71 are detected by cell culture or PCR assay, collected from respiratory droplets like saliva, sputum, nasal mucus, and faecal material of infected persons. In the year 2019, Alexandra et al. found that EV71 can enter the host body through the faecal–oral route and initially target the gastrointestinal epithelium than the respiratory tract. After that, it gradually infects the host body [13].

## Main text

### Methodology

A basic and thorough overview of the literature surveyed to identify the viral infection of EV71 was conducted till 2021. Many offline and online databases were taken into consideration. The review articles and research papers published by various reputed publishers such as Elsevier, Springer, Taylor & Francis imprints, and Hindawi were considered as the primary source of data collection for this review article. Some online databases including NCBI, PubMed, Google Scholar, ProQuest, Scopus, and EBSCO were also accessed using keywords relating to the topic for data mining. The paid articles were accessed through the Centre Library facility of Siksha O Anusandhan University. The conference proceedings, magazines, WebPages, and book chapters were also reviewed and accessed as the other sources of the literature to maximize the information about the current bottlenecks, the extent of research carried out, and the potential utility of the topic. In this review, it is discussed about the structure, outbreak, and molecular epidemiology of Enterovirus 71 along with different EV71 vaccines. The EV71 infection is a major threat to public health across the world. The infections are mostly seen in children and very rarely in adults.

## Structure of the EV71

The structure of Enterovirus 71 is a single-stranded RNA virus with an RNA genome size of around 7.4 kb [14]. Folegatti et al. [15] reported that different enteroviruses have a distinct structure, sequence, genome, and biological activity. The EV71 is non-enveloped and icosahedral with a diameter of about 20–30 nm. Also, the coding area of EV71 is divided into 3 sections such as P1, P2, and P3. The P1 is encoded with 4 structural viral proteins like VP1, VP2, VP3, and VP4. Similarly, P2 is encoded with three non-structural proteins like 2A, 2B, and 2C, whereas P3 is encoded with four non-structural proteins like 3A, 3B, 3C, and 3D. The P2 and P3 are combined to form the proteases that are used in proteolytic cleavage for the production of structural proteins.

Moreover, the capsid of EV71 has 60 copies of VP1, VP2, VP3, VP4, and the structural proteins are combined to make a protomer. Further, 5 protomers form a pentamer and 12 pentamers are combined to make a virion [16, 17]. In contrast, VP1, VP2, and VP3 are present in the outer layer of the viral capsid, and the host immune system is affected by these 3 proteins. Moreover, some neutralizing epitopes of VP1 are used as biomarkers for vaccines [18]. Genotype A has the prototype strain (Br Cr), while B has 5 sub-genotypes (B1 to B5), and C has five sub-genotypes like C1, C2, C3, C4, and C5. The EV71 is dependent on VP1. Moreover, an untranslated region (UTR) is present at the 5' and 3' end of the RNA genome. The 5' UTR has an internal ribosomal entry site for cap-independent translation, and it is bound with VPg (viral protein), while 3'UTR possesses a poly-A tail. The RNA translated to polyprotein is consecutively cut by the viral 2A protease, 3D protease, and 3C protease. Viral protein 3D shows RNA-dependent RNA polymerase activity.

**Fig. 1:**
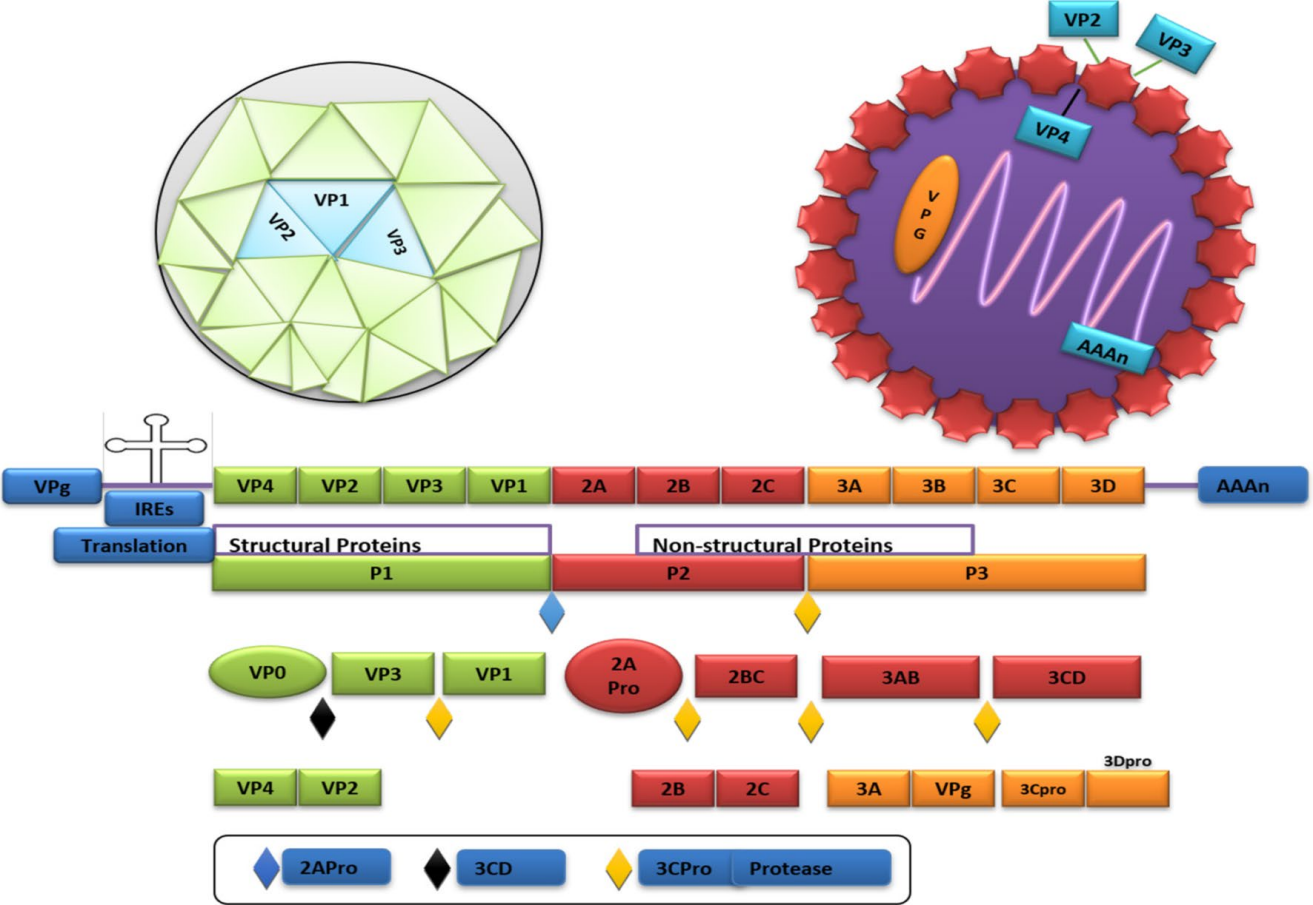
Structural representations of EV71

Further, the Picornaviridae viral genomes are composed of IRES. So, the virus can enter the host cells and release the viral genome in the cytoplasm, and IRES can be interpreted by viral RNA. At the time of viral translation and replication, the virus needs IRES-specific trans-acting factors (ITAFs). It requires some other factors like T cell-restricted intracellular antigen 1 (TIA-1) and TIA-1-related protein (TIAR) for viral translation and replication. The interaction of TIA-1 and TIAR with the 5´ untranslated region of the viral genome can increase the replication [19]. The structure of EV71 is shown in Fig. 1.

## Categories of enteroviruses

Enteroviruses are classified into several types as poliovirus (PV) is a micro, unenveloped virus of the genus Enterovirus, with a single-stranded genomic positive ribonucleic acid of approx. 7500nt Picornaviridae family. The PV is a causal factor in poliomyelitis, where motor neurons are the primary target. The PV motor neuron tropism is due in part to PVR (PV receptor) expression. In this regard, EV71 relates to the EV community, but has been split as PV, human EV genus A, into many genera. In addition to, Enterovirus 68 has induced lower lung infections in the younger forms of Enterovirus 70, that is an agent of extreme acute haemorrhagic conjunctivitis epidemics, where EV71 (Enterovirus 71) has exacerbated aseptic meningitis and hand, foot, and mouth disease (HFMD) and encephalitis in a variety of nations. Aseptic meningitis, herpangina, outbreak myalgia (pleurodynia, Bornholm syndrome), hand, foot, and mouth illness, myocarditis, pericarditis, diarrhoea, rashes, and sinus infections are signs with several diseases. In specific congenital malformations and maybe in some instances of diabetes, they can also play a part. Among all, the echovirus-induced diseases are aseptic meningitis, febrile infections without or with a cough, and rash. Table 1 describes different categories of EV71 [20].

**Table 1:** Different categories of Enteroviruses (EV71)

SL. No	Virus	Serotypes	Clinical diseases
1	Poliovirus	3 types	Asymptomatic infection, viral meningitis, paralytic disease, poliomyelitis
2	Coxsackie A viruses	23 types (A1-A22, A24)	Viral meningitis plus, rash, ARD, myocarditis, orchitis
3	Coxsackie B viruses	6 types (B1-B6)	Viral meningitis, but no orchitis
4	Echoviruses	32 types	Viral meningitis, with orchitis
5	Other enteroviruses	4 types (68–71)	Viral meningitis

## EV71 Receptors bind with host body

**Fig. 2:**
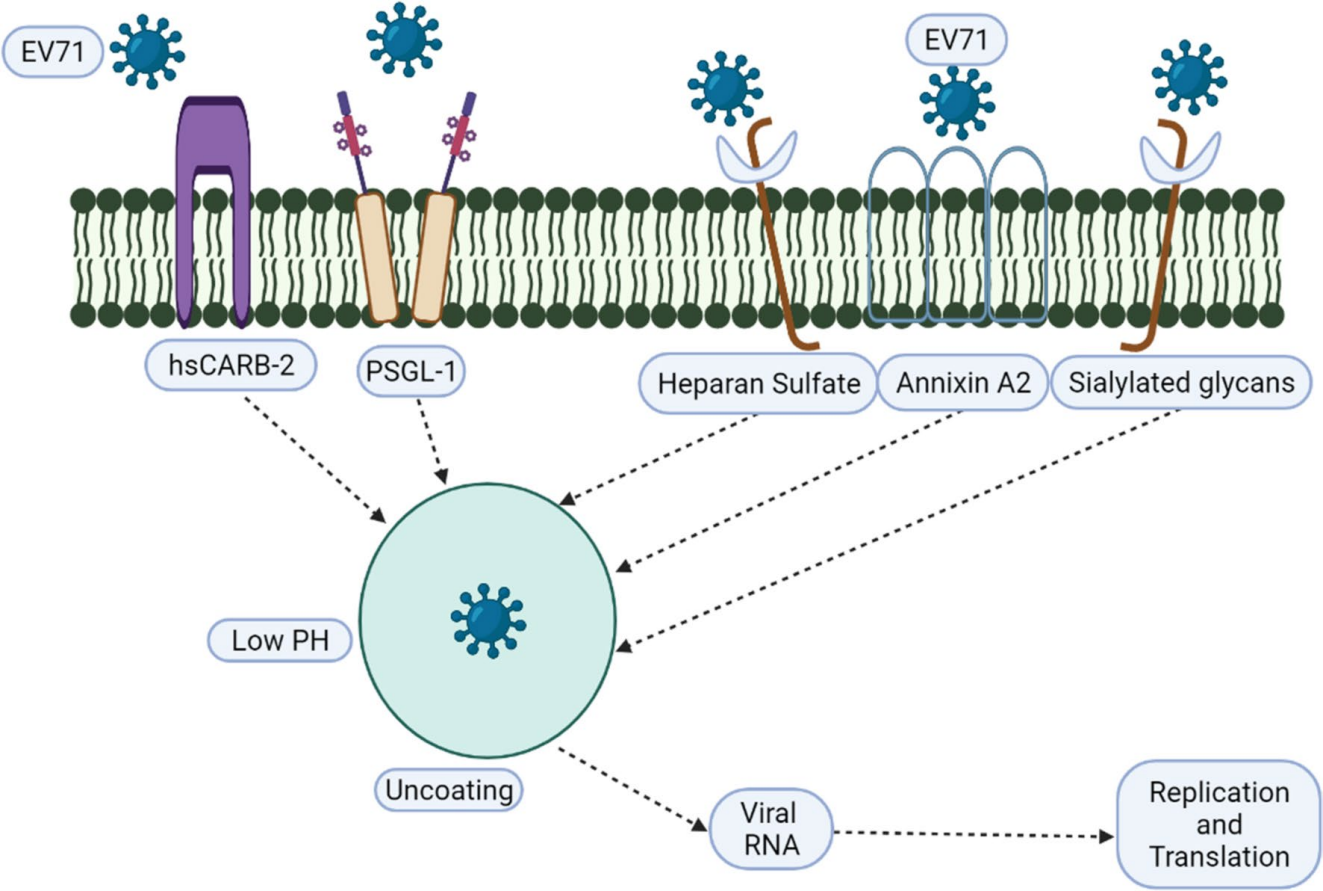
EV71 viral receptors binding process with host body

Some important EV71 viral receptors like P-selectin glycoprotein ligand 1 (PSGL-1), human scavenger receptor (hSCARB2), human dendritic cell-specific intercellular adhesion molecule-3 grabbing nonintegrin (DC-SIGN), annexin A2 (AnxA2), heparan sulphate (HS), and sialylated glycan have the ability to bind with the host cells [21]. After binding the viral receptor with the host cell, the viral coat is dissociated and the RNA is released. The protein translation and viral replication start in the host cell as shown in Fig. 2 [22].

## The outbreak of EV71 in different countries

Initially, HFMD was detected in April 1957 with 8 children infected in New Zealand and during the 1960s the major outbreak of HFMD occurred by the CVA16 virus [23]. In 1969, EV71 was detected in the USA in a 9-month baby with encephalitis. The virus also was identified in California, and after that, it spread across the world [24]. In 1997, the first major outbreak of HFMD occurred in the Asia–Pacific region.

In 1981, HFMD was first identified in Shanghai, China. Later, in 2008, approximately 488,955 cases were detected with a mortality rate of 126 in China [25, 26]. Between 2008 and 2012, around 7,200,092 infected patients were identified with a death rate of 2,457, which was announced by the Chinese Centre for Disease Control and Prevention. EV71 was an epidemic with the C4 genotype in China [27]. On 3 May 2008, Chinese health authorities informed that the outbreak of EV71 mostly occurred in specific areas like Fuyang, Anhui, Zhejiang, and Guangdong. In 2008, total 3,3736 cases were detected, and among them 22 patients were dead, while 42 were severely complicated cases. Approximately, 415 patients were detected in 24 h in Fuyang. On 5 May 2008, around 6,300 patients were detected in Zhejiang with a death rate of 26, and 5,151 patients were detected in Anhui, while 8,531 cases with hand–foot–mouth disease were detected in China, where age of the affected infants was less than 6 or 2. In Taiwan, in the year 1998, around 129,101 infected patients with HFMD were detected, and among them, 405 patients were found suffering from neurological disorders like meningitis and encephalitis with a mortality rate of 78. In 2008, 387 serious patients were identified with EV71 by HFMD, and the mortality rate was 14 in Taiwan [28]. In Taiwan, EV71 was identified in 44.4% of patients in the year 1998, 2% in 1999, and 20.5% in 2000.

Most EV71 cases detected in 1998 were with genotype C, while 1/10 was identified with genotype B. The death rate of EV71 was 78 in 1998, 25 in 2000, and 26 in 2001. Lin and Tzou categorized 1998 outbreak of Taiwan into 2 phases. The first phase was witnessed on 7 June, with around 405 critical cases, and 91% of infants below 5 years old were dead [29]. Further, it was found that 16% of cases were seen in six months or younger children, while 43% were 7–12 months children, and 65 out of 78 children lost lives due to pulmonary oedema or haemorrhage, which are associated with the lethal virus. The second phase occurred on 4 October with very few cases, which were detected in Southern Taiwan. In 2003, first EV71 infected child was identified in Vietnam with HFMD between 2011 and 2012, and around 200,000 cases were detected with a mortality rate of 207. Approximately, 63,780 cases were detected in the first 7 months of 2012 with HFMD [30].

Tran Minh Dien, vice director of the National Hospital for infants, announced that 58.7% of cases were occurred by EV71 in Vietnam. In South Korea, the first infected patient with HFMD was recognized in spring 2009 and hospitalized; after some days it was found that all the patients were dead owing to severe complications. Besides these, HFMD occurred in Japan in 1997–2000 and Singapore and Malaysia in the year 1997. Chen et al. demonstrated that EV71 is mostly seen in Asia–Pacific countries. Moreover, the C4 genotype of EV71 is currently found in China, Hong Kong, Korea, and Vietnam, while B5 was found in Japan, Malaysia, and Taiwan.

In Australia, in June 2013 around 100 children were infected by EV71, of which four cases were detected in Sydney. In Cambodia, between April and July 2012, the fatality rate was 64, and 2 survived, who were under 7 years old. Infected children died in between 24 h, and symptoms were mainly respiratory problems, fever, and neurological abnormalities. On 6 July 2012, out of 24, 15 were found positive with EV71. After that, the infection rate gradually decreased. On 15 July 2012, WHO declared that Cambodia is free from EV71 [31].

## Molecular epidemiology

The Enterovirus 71 genotypes are detected in different countries, as shown in Table 2. In the year 1969, a child in the USA was detected EV-A71 with encephalitis [32]. After that, the infections got spread rapidly in the 1970s among the children of America, Europe, and Australia [33, 34]. In 2017, Shin et al. reported that the EV-A71 is closed by capsid proteins VP1, VP2, VP3, and VP4, where VP1 has antigenicity and neutralization factor. Further, Yi et al. reported that based on the VP1 nucleotide sequence, EV-A71 can be classified as 3 separate genogroups such as A, B, and C [35, 36]. Genogroup A contains the prototype EV-A71 strain (BrCr-CA-70) and first detected in the USA in 1969, but in China, it was not detected until 2008.

**Table 2:** EV-A71 genotypes in different countries

Countries	1960–1969 year	1970–1979 year	1980–1989 year	1990–1999 year	2000–2009 year	2010–2016 year
Singapore				B3, B4	B4, B5, C1	
Malaysia				B3, B4	B4, B5, C1	
Australia				B3, C2	C1	
Japan				B3, B4, C2	B4, B5, C2, C4a	C2
Korea				B4, C2	C2, C3, C4a, C4b	C4a
Taiwan				B4, C2	B4, B5, C4, C5	B5, C4
China					C4	C4, C4a
Cambodia						C4
Vietnam						C4, B5
France					C1, C2, C4	C4
UK				C1	C1, C2	
Germany					C1, C2	
Austria					C1, C4	
Norway					C1	
Netherlands	B0	B1	B1	C1	C1, C2	
Hungary		B1			C1, C4	
Bulgaria		B1				
USA	A	B1	B1	C1, C2	C2	
Peru					C1	

According to Solomon et al. [4] genogroup B is categorized into sub-genogroups, i.e. B1, B2, B3, B4, B5. Further, genogroup C is also classified into sub-genogroups C1, C2, C3, C4, and C5 [37, 38]. Moreover, the C4 genogroup is again subclassified into the C4a and C4b lineages. However, genogroup D was first detected in India, and genogroups E and F were first recognized in Africa [39]. In addition, EV71 is seen more in the Asia–Pacific region [40]. In the Asia–Pacific region, genogroup A was not detected until 2008, but genogroups B and C were identified since 1997. Moreover, the subgroups like B3, B4, C1, and C2 rapidly spread in this area since 1990–2016. Also, C4 and C4a were circulating in this area, where the nucleotide and amino acid mutations of C4a are relatively found the same as C4b.

Further, it was found that the change of C4a to C4b is the major reason for increasing neurovirulence epidemics in China [41, 42]. From the genetic and antigenic analysis, it was reported that C4a was spreading not only from China to Vietnam, but also highly spread in Ho Chi Minh City, Southern Vietnam, in 2011. Bible et al. reported that EV-A71 strains are also spreading outside the Asia–Pacific region [43]. From 1963 to 1986, B0, B1, and B2 subgroups were identified in the Netherlands [44]. Moreover, during the year 1987, B genogroup was replaced by C, C1, and C2 [45]. Based on the epidemiological study, subgroups B1, B2, C1, and C2 gradually spread its tentacles in Europe, Australia, and the USA [46]. In addition to this, B3-B5, C4, and C5 were seen in the Asia–Pacific region from 1997, but not seen outside of that area. Gradually, EV-A71 started spreading across the world [47]. From 1900 to 2016, infections were rapidly spread by sub-genogroup B0, B1 and B2, C2, C3, C4a, and C4b. From the basis of molecular epidemiological study, it was found that mutated EV-A71 spread across the world, while the sub-genogroup B5 had a various antigenicity from B1, B4, C2, and C4 [47].

## Clinical symptoms in EV71

The most frequently observed disease in EV71-infected patients is pyrexia (eighty-one per cent) with a skin rash (seventy per cent), insomnia (sixty-six per cent), vomiting (sixty-two per cent), lethargy (forty per cent), myoclonus (sixty per cent), and ataxia (forty-two per cent). CSF (cerebrospinal fluid) evaluations were carried out in 47 clients, where 45 were pleocytosis, 94/ml median, 15–920/ml range among them. However, none of them had viruses isolated from specimens of CSF. The most frequently diagnosed vector of CA16 was HFMD, while CA5, CA9, and CA10 were statically identified as HFMD as a source among other recognized HFMD enteroviruses.

The EV71 and CA16 are highly neurotropic viruses that have caused severe CNS issues in patients, and these CNS complications have been identified as the primary causes of fatal HFMD. From the clinical experiments in Western Australia, and Japan, Malaysia indicate that hand–foot–mouth disease (HFMD) rashes may be variable because of CA16 and EV71. Rashes or erythema occurs due to CA16 in the arms and legs, of larger vesicles than the EV71 virus, where the rash is most commonly popular and petechial in those viruses. The infection of three to four days was characterized, as the disorder in the mouth, gums, palate, papulosicles and the distribution of vesicular enanthemum in the labia, buttocks, feet seen in EV71 patients. Some major clinical symptoms are briefly shown in Table 3 [48, 49].

**Table 3:** The Clinical signs in EV71 patients

SL No	Sign/Symptom	Total no. of infected patients (%)	EV71 infected patients (%)
1	Fever (°C)	81	43
2	Fever (37–38 °C)	8	4
3	Fever (38–39 °C)	34	18
4	Fever (> 39 °C)	40	21
5	Skin eruption	70	37
6	Vomiting	62	33
7	Myoclonus	60	32
8	Sleep disturbance	66	35
9	Lethargy	40	21
10	Ataxia	42	22
11	Neck stiffness	15	8
12	Headache	21	11
13	Apathy	9	5
14	Nystagmus	11	6

## Pathogenesis

**Fig. 3:**
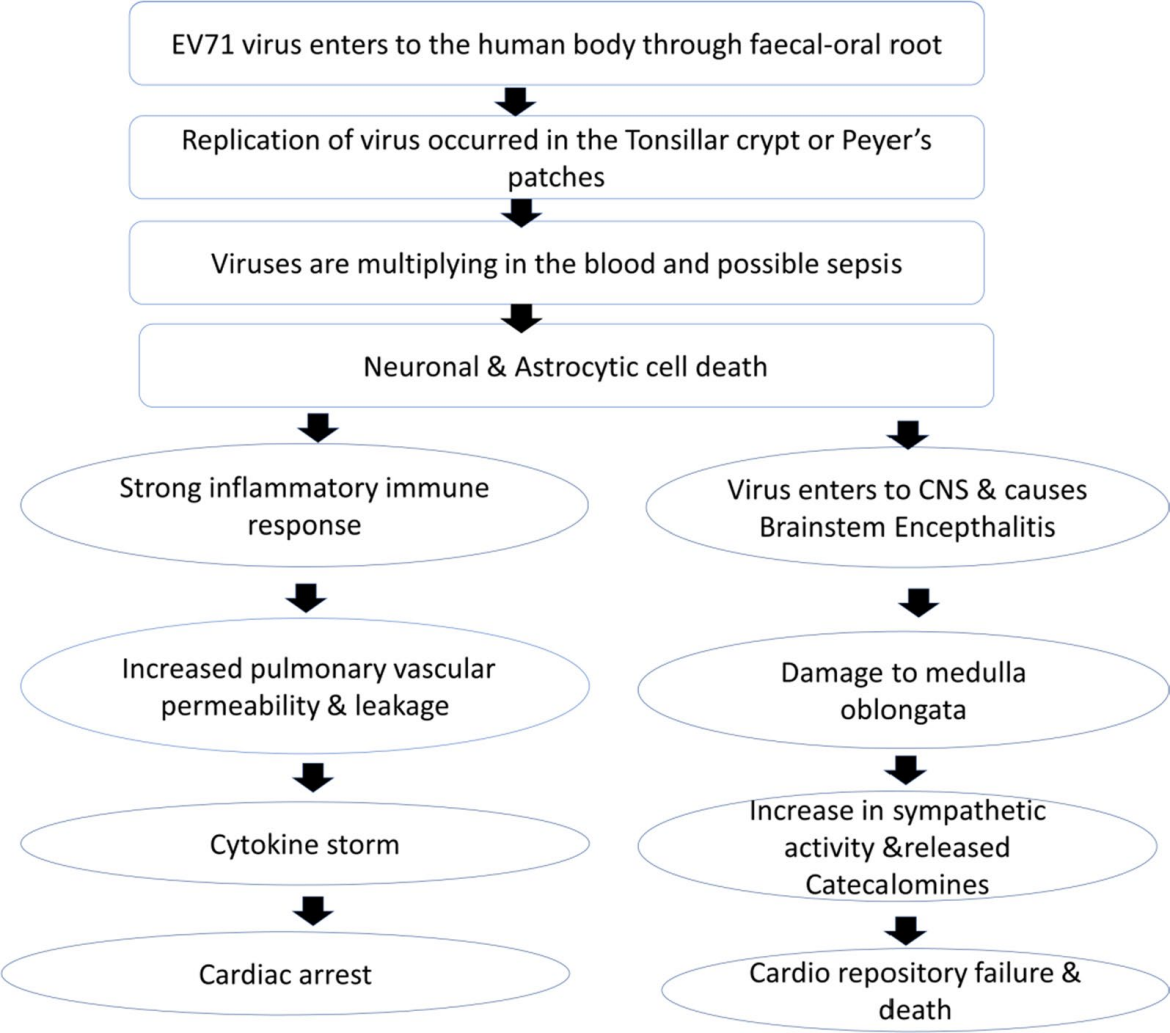
Flow chart of pathogenesis of Enterovirus 71

The flow chart of the pathogenesis of Enterovirus 71 is presented in Fig. 3. Enterovirus 71 mostly occurred in brainstem encephalitis that specifically affects the medulla and is connected with cardiopulmonary dysfunction. EV71 infection is more effective in Asia with an excessive death rate. EV71 causes a highly burning illness that persists for 3–10 days. HFMD transmission can be controlled not only by isolating patients, but also by maintaining proper hygiene, like hand-washing in regular intervals and frequently cleaning the surfaces of accessible objects [50]. This virus causes serious health hazards and leads to death. EV71 is a neurotropic virus, and the main target is to affect the brain stem [51]. EV71 can enter the CNS through two routes. The virus can either enter the CNS by the blood–brain barrier or by peripheral nerves through retrograde axonal transport [52]. EV71 virus also transmits to the CNS by peripheral motor nerves, and the skeletal muscle gets immediately infected by the CNS not only by motor neurons but also by other neural pathways.

From the study of autopsy in Malaysia, inflammation was found in the spinal cord grey matter, brainstem, hypothalamus, subthalamic and dentate nuclei [53]. Neurological virulence is reported to be a major cause of death [54]. Li et al. [55] suggested that an L97R alteration in the VP1 protein increases the neuronal tropism of EV71, although the alterations in VP1, 5’ NCR, and protease 2A affect viral virulence [55, 56]. The change in variation in the sequences in RNA causes some neurological infections. The mechanism of action of pulmonary oedema of EV71 infection is the destruction of the medial, ventral, and caudal medullas, which causes blood shift to the lungs [57]. Moreover, pulmonary oedema is also developed in children suffering from EV71 brainstem encephalitis.

It has also been reported that abnormal cytokine activation produces severe inflammation in the lungs [58]. From several studies, it was found that children suffering from severe EV71 encephalitis have cytotoxic T lymphocyte antigen haplotype (CTLA-4). It is also reported that EV71 causes aseptic meningitis and fever which are adverse health effects in children [59]. Based on autopsy studies, it was found that the mortality rate of EV71 infection in Taiwan and Hong Kong had occurred by brainstem encephalitis. Enteroviruses not only get influenced by viral factors but also proliferate host factors in their life cycle. The interaction of the viral and host factors plays a key role in viral replication. Although tissue-specific viral virulence is poorly understood in cell-based systems and animal models, it needs more study in the future [60].

## EV71 vaccines

Some EV71 infections are not only symptomatic but also asymptomatic and can be controlled by vaccine [61]. Till date, different EV71 vaccines have been developed such as inactivates virus vaccine and virus-like particle vaccine, DNA vaccines subunit vaccine also a live attenuated vaccine [62, 63]. From the previous study, it was reported that inactivated whole-virus EV71 vaccines are more effective as compared to other inactivated vaccines [64]. From the previous occurrence, it was also found that formaldehyde-inactivated EV71 vaccines are very effective and protective for any animal model.

On the other hand, a formalin-inactivated EV71 vaccine is expressed as an alum-adjuvant vaccine which causes cross-neutralizing antibody comebacks in a phase III trial. As per reports, about 30,000 children in China were cured in phase III clinical trials of inactivated EV71 vaccines. In December 2015, China’s Food and Drug Administration officially investigated 2 inactivated EV-A71 vaccines which inhibited serious HFMD in children. On the other hand, formalin-inactivated EV71 vaccines were developed in Taiwan and Singapore which underwent Phase I clinical trials, and the results were excellent for preventing of EV71 infection. Although inactivated EV71 vaccines were mostly used for safeguarding the children, they had the potential to inhibit the replication of the viral genome.

The virus-like particles (VLPs) vaccines are completely different from the inactivated whole-virus EV71 vaccines. VLPs vaccine possesses all surface epitopes of the capsid protein and is very less effective than inactivated vaccines [65]. In addition to this, some new EV71 vaccines such as DNA vaccines, subunit vaccines, and peptide vaccines have been developed. EV71 vaccine development is active and ongoing in Asian nations. Vaccine researchers and developers in both developed and developing countries must work together to develop a safe and effective EV71 vaccine.

## Conclusions

EV71 is a life-threatening disease for infants and children across the world. This viral infection is fast-spreading in Asia, causing chronical health hazards like HFMD, neurological abnormalities among children. HFMD is a viral and transmissible disease. Children get infected in 3 days after exposure to the virus and remain infectious until 10 days. The major symptoms of this infection are fever, runny nose, sneezing, cough, rashes in skin, ulcer in mouth and muscle aches, etc.

To date, no specific treatments have been found for EV71 virus. Children with mild symptoms are one of the reasons for non-polio enterovirus infection. Most of the children recover completely, but some get affected with serious complications or lethal to the virus. Therefore, there is an urgency of treatment for these viral infections. EV71 vaccination is a priceless gift for children in Asia–Pacific as well as across the world. Clinical studies on inactivated EV71 vaccines have proven to be a useful weapon in the battle against EV71 infection-associated severe HFMD outbreaks. Companies in Asia lead the development of EV71 vaccines, demonstrating that vaccine R&D and assessment in these Asian nations have improved. Nonetheless, market entrance approvals and applications in other countries with significant targeted populations of babies and children face technological and legislative obstacles. Vaccine availability, the possibility of introducing the EV71 vaccination into EPI, and the harmonization of vaccine standards are the hurdles. As a result, international collaboration is critical for controlling the spread of the EV71 vaccination in underdeveloped countries. WHO should take the lead in developing EV71 vaccine quality control and assessment standards.

The good news is that the National Institutes of Health's National Institute of Food and Drug Control and the National Institute of Biological Standards and Control are working together to develop WHO neutralizing antibody standards for EV71 vaccines, which could help standardize vaccine quality control and evaluation. Only the Japanese encephalitis (JE) vaccine developed by the Chengdu Institute in China has received WHO pre-qualification. The world still has a long way to go before the cost-effective EV71 vaccine is widely available.

## Data Availability

Not applicable.

## Abbreviations

EV71: Enterovirus 71
HFMD: Hand–foot–mouth diseases
CNS: Central nervous system
WHO: World Health organization
VLPs: Virus-like particles
VP: Viral protein
IRES: Internal ribosome entry site
ITAFs: IRES-specific trans-acting factors
TIA-1: T cell-restricted intracellular antigen 1
PSGL-1: P-selectin glycoprotein ligand 1
hSCARB2: Human scavenger receptor
DC-SIGN: Dendritic cell-specific intercellular adhesion molecule-3 grabbing nonintegrin
AnxA2: Annexin A2
HS: Heparan sulphate
CTLA-4: Cytotoxic T-lymphocyte-associated antigen 4
CA: Coxsackievirus A
CB: Coxsackievirus B

## References

[1] Goksugur N, Goksugur S (2010). Images in clinical medicine hand, foot, and mouth disease. N Engl J Med.

[2] Omaña-Cepeda C, Martínez-Valverde A, del Mar S-R, Jané-Salas E, Marí-Roig A, López-López J (2016). A literature review and case report of hand, foot and mouth disease in an immunocompetent adult. BMC Res Notes.

[3] Zhou XN, Zhang XZ, Wang B, Qiu YT (2012). Etiologic and epidemiologic analysis of hand, foot, and mouth disease in Guangzhou city: a review of 4,753 cases. Braz J Infect Dis.

[4] Solomon T, Lewthwaite P, Perera D, Cardosa MJ, McMinn P, Ooi MH (2010). Virology, epidemiology, pathogenesis, and control of enterovirus 71. Lancet Infect Dis.

[5] Kaminska K, Martinetti G, Lucchini R, Kaya G, Mainetti C (2013). Coxsackievirus A6, and hand, foot and mouth disease: three case reports of familial child-to-immunocompetent adult transmission and a literature review. Case Rep Dermatol.

[6] Chumakov M, Voroshilova M, Shindarov L, Lavrova I, Gracheva L, Koroleva G, Vasilenko S, Brodvarova I, Nikolova M, Gyurova S, Gacheva M, Mitov G, Ninov N, Tsylka E, Robinson I, Frolova M, Bashkirtsev V, Martiyanova L, Rodin V (1979). Enterovirus 71 isolated from cases of the epidemic poliomyelitis-like disease in Bulgaria. Arch Virol.

[7] Kim SJ, Kim JH, Kang JH, Kim DS, Kim KH, Kim KH, Kim YH, Chung JY, Bin JH, Jung DE, Kim JH, Kim HM, Cheon DS, Kang BH, Seo SY (2013). Enteroviruses complications working group. Risk factors for neurologic complications of hand, foot, and mouth disease in the Republic of Korea, 2019. J Korean Med Sci.

[8] Zhou F, Kong F, Wang B, McPhie K, Gilbert GL, Dwyer DE (2011). Molecular characterization of enterovirus 71 and coxsackievirus A16 using the 5' untranslated region and VP1 region. J Med Microbiol.

[9] Schubert RD, Hawes IA, Ramachandran PS, Ramesh A, Crawford ED, Pak JE, Wu W, Cheung CK, O'Donovan BD, Tato CM, Lyden A, Tan M, Sit R, Sowa GM, Sample HA, Zorn KC, Banerji D, Khan LM, Bove R, Hauser SL, Gelfand AA, Johnson-Kerner BL, Nash K, Krishnamoorthy KS, Chitnis T, Ding JZ, McMillan HJ, Chiu CY, Briggs B, Glaser CA, Yen C, Chu V, Wadford DA, Dominguez SR, Ng TFF, Marine RL, Lopez AS, Nix WA, Soldatos A, Gorman MP, Benson L, Messacar K, Konopka-Anstadt JL, Oberste MS, DeRisi JL, Wilson MR (2019). Pan-viral serology implicates enteroviruses in acute flaccid myelitis. Nat Med.

[10] Chen KT, Chang HL, Wang ST, Cheng YT, Yang JY (2007). Epidemiologic features of hand-foot-mouth disease and herpangina caused by enterovirus 71 in Taiwan, 1998–2005. Pediatrics.

[11] Chen YJ, Meng FY, Mao Q, Li JX, Wang H, Liang ZL, Zhang YT, Gao F, Chen QH, Hu Y, Ge ZJ, Yao X, Guo HJ, Zhu FC, Li XL (2014). Clinical evaluation for batch consistency of an inactivated enterovirus 71 vaccines in a large-scale phase 3 clinical trial. Hum Vaccin Immunother.

[12] Lee MS, Chang LY (2010). Development of enterovirus 71 vaccines. Expert Rev Vaccines.

[13] Alexander JP, Baden L, Pallansch MA, Anderson LJ (1994). Enterovirus 71 infections and neurologic disease in the United States, 1977–1991. J Infect Dis.

[14] Wang SM, Ho TS, Lin HC, Lei HY, Wang JR, Liu CC (2012). Reemerging of enterovirus 71 in Taiwan: the age impact on disease severity. Eur J Clin Microbiol Infect Dis.

[15] Folegatti PM, Ewer KJ, Aley PK, Angus B, Becker S, Belij-Rammerstorfer S, Bellamy D, Bibi S, Bittaye M, Clutterbuck EA, Dold C, Faust SN, Finn A, Flaxman AL, Hallis B, Heath P, Jenkin D, Lazarus R, Makinson R, Minassian AM, Pollock KM, Ramasamy M, Robinson H, Snape M, Tarrant R, Voysey M, Green C, Douglas AD, Hill AVS, Lambe T, Gilbert SC, Pollard AJ (2020). Oxford COVID vaccine trial group safety and immunogenicity of the ChAdOx1 nCoV-19 vaccine against SARS-CoV-2: a preliminary report of a phase 1/2, single-blind, randomized controlled trial. Lancet.

[16] Ma HC, Liu Y, Wang C, Strauss M, Rehage N, Chen YH, Altan-Bonnet N, Hogle J, Wimmer E, Mueller S, Paul AV, Jiang P (2014). An interaction between glutathione and the capsid is required for the morphogenesis of C-cluster enteroviruses. PLoS Pathog.

[17] Rossmann MG, Arnold E, Erickson JW, Frankenberger EA, Griffith JP, Hecht HJ, Johnson JE, Kamer G, Luo M, Mosser AG (1985). Structure of a human common cold virus and functional relationship to other picornaviruses. Nature.

[18] Lin JY, Shih SR, Pan M, Li C, Lue CF, Stollar V, Li ML (2009). hnRNP A1 interacts with the 5' untranslated regions of enterovirus 71 and Sindbis virus RNA and is required for viral replication. J Virol.

[19] Yang SL, Chou YT, Wu CN, Ho MS (2011). Annexin II binds to capsid protein VP1 of enterovirus 71 and enhances viral infectivity. J Virol.

[20] Li Y, Chang Z, Wu P, Liao Q, Liu F, Zheng Y, Luo L, Zhou Y, Chen Q, Yu S, Guo C (2018). Emerging enteroviruses causing hand, foot and mouth disease. China Emerg Infect Dis.

[21] Ren XX, Ma L, Liu QW, Li C, Huang Z, Wu L, Xiong SD, Wang JH, Wang HB (2014). The molecule of DC-SIGN capture enterovirus 71 and confers dendritic cell-mediated viral trans-infection. Virol J.

[22] Ruan F, Yang T, Ma H, Jin Y, Song S, Fontaine RE, Zhu BP (2011). Risk factors for hand, foot, and mouth disease and herpangina and the preventive effect of hand-washing. Pediatrics.

[23] Duff MF (1968). Hand-foot-and-mouth syndrome in humans: coxsackie A10 infections in New Zealand. Br Med J.

[24] Chong P, Liu CC, Chow YH, Chou AH, Klein M (2015). Review of enterovirus 71 vaccines. Clin Infect Dis.

[25] Wang X, Peng W, Ren J, Hu Z, Xu J, Lou Z, Li X, Yin W, Shen X, Porta C, Walter TS, Evans G, Axford D, Owen R, Rowlands DJ, Wang J, Stuart DI, Fry EE, Rao Z (2012). A sensor-adaptor mechanism for enterovirus uncoating from structures of EV71. Nat Struct Mol Biol.

[26] Cho HK, Lee NY, Lee H, Kim HS, Seo JW, Hong YM, Lee SJ, Lee SW, Cheon DS, Hong JY, Kang BH, Kim JH (2009). Kim KH (2010) Enterovirus 71-associated hand, foot and mouth diseases with neurologic symptoms, a university hospital experience in Korea. Korean J Pediatr.

[27] Chen B, Sumi A, Toyoda S, Hu Q, Zhou D, Mise K, Zhao J, Kobayashi N (2015). Time series analysis of reported cases of hand, foot, and mouth disease from 2010 to 2013 in Wuhan. China BMC Infect Dis.

[28] Huang SW, Hsu YW, Smith DJ, Kiang D, Tsai HP, Lin KH, Wang SM, Liu CC, Su IJ, Wang JR (2009). Reemergence of enterovirus 71 in 2008 in Taiwan: dynamics of genetic and antigenic evolution from 1998 to 2008. J Clin Microbiol.

[29] Lin TY, Twu SJ, Ho MS, Chang LY, Lee CY (2003). Enterovirus 71 outbreaks, Taiwan: occurrence and recognition. Emerg Infect Dis.

[30] Donato C, le Hoi T, Hoa NT, Hoa TM, Van Duyet L, Dieu Ngan TT, Van Kinh N, Vu Trung N, Vijaykrishna D (2016). Genetic characterization of enterovirus 71 strains circulating in Vietnam in 2012. Virology.

[31] Kua JA, Pang J (2020). The epidemiological risk factors of hand, foot, mouth disease among children in Singapore: A retrospective case-control study. PLoS ONE.

[32] Schmidt NJ, Lennette EH, Ho HH (1974). An apparently new enterovirus isolated from patients with disease of the central nervous system. J Infect Dis.

[33] Geoghegan JL, le Tan V, Kühnert D, Halpin RA, Lin X, Simenauer A, Akopov A, Das SR, Stockwell TB, Shrivastava S, Ngoc NM, le Uyen TT, Tuyen NT, Thanh TT, Hang VT, Qui PT, Hung NT, Khanh TH, le Thinh Q, le Nhan NT, Van HM, Viet do C, Tuan HM, Viet HL, Hien TT, Chau NV, Thwaites G, Grenfell BT, Stadler T, Wentworth DE, Holmes EC, Van Doorn HR (2015). Phylodynamics of enterovirus A71-associated hand, foot, and mouth disease in Viet Nam. J Virol.

[34] Witsø E, Palacios G, Rønningen KS, Cinek O, Janowitz D, Rewers M, Grinde B, Lipkin WI (2007). Asymptomatic circulation of HEV71 in Norway. Virus Res.

[35] Yi EJ, Shin YJ, Kim JH, Kim TG, Chang SY (2017). Enterovirus 71 infection and vaccines. Clin Exp Vaccine Res.

[36] Ortner B, Huang CW, Schmid D, Mutz I, Wewalka G, Allerberger F, Yang JY, Huemer HP (2009). Epidemiology of enterovirus types causing neurological disease in Austria 1999–2007: detection of clusters of echovirus 30 and enterovirus 71 and analysis of prevalent genotypes. J Med Virol.

[37] Zhang Y, Zhu Z, Yang W, Ren J, Tan X, Wang Y, Mao N, Xu S, Zhu S, Cui A, Zhang Y, Yan D, Li Q, Dong X, Zhang J, Zhao Y, Wan J, Feng Z, Sun J, Wang S, Li D, Xu W (2010). An emerging recombinant human enterovirus 71 responsible for the 2008 outbreak of hand foot and mouth disease in Fuyang city of China. Virol J.

[38] Tee KK, Lam TT, Chan YF, Bible JM, Kamarulzaman A, Tong CY, Takebe Y, Pybus OG (2010). Evolutionary genetics of human enterovirus 71: origin, population dynamics, natural selection, and seasonal periodicity of the VP1 gene. J Virol.

[39] Bessaud M, Razafindratsimandresy R, Nougairède A, Joffret ML, Deshpande JM, Dubot-Pérès A, Héraud JM, de Lamballerie X, Delpeyroux F, Bailly JL (2014). Molecular comparison and evolutionary analyses of VP1 nucleotide sequences of new African human enterovirus 71 isolates reveal a wide genetic diversity. PLoS ONE.

[40] Hosoya M, Kawasaki Y, Sato M, Honzumi K, Kato A, Hiroshima T, Ishiko H, Suzuki H (2006). Genetic diversity of Enterovirus 71 associated with hand, foot, and mouth disease epidemics in Japan from 1983 to 2003. Pediatr Infect Dis J.

[41] Hsia SH, Wu CT, Chang JJ, Lin TY, Chung HT, Lin KL, Hwang MS, Chou ML, Chang LY (2005). Predictors of unfavorable outcomes in enterovirus 71-related cardiopulmonary failure in children. Pediatr Infect Dis J.

[42] Zhang Y, Wang J, Guo W, Wang H, Zhu S, Wang D, Bai R, Li X, Yan D, Wang H, Zhang Y, Zhu Z, Tan X, An H, Xu A, Xu W (2011). Emergence and transmission pathways of rapidly evolving evolutionary branch C4a strains of human Enterovirus 71 in the Central Plain of China. PLoS ONE.

[43] Bible JM, Iturriza-Gomara M, Megson B, Brown D, Pantelidis P, Earl P, Bendig J, Tong CY (2008). Molecular epidemiology of human Enterovirus 71 in the United Kingdom from 1998 to 2006. J Clin Microbiol.

[44] Lukashev AN, Shumilina EY, Belalov IS, Ivanova OE, Eremeeva TP, Reznik VI, Trotsenko OE, Drexler JF, Drosten C (2014). Recombination strategies and evolutionary dynamics of the human Enterovirus a global gene pool. J Gen Virol.

[45] Chakraborty R, Iturriza-Gómara M, Musoke R, Palakudy T, D'Agostino A, Gray J (2004). An epidemic of Enterovirus 71 infections among HIV-1-infected orphans in Nairobi. AIDS.

[46] Schuffenecker I, Mirand A, Antona D, Henquell C, Chomel JJ, Archimbaud C, Billaud G, Peigue-Lafeuille H, Lina B, Bailly JL (2011). Epidemiology of human Enterovirus 71 infections in France, 2000–2009. J Clin Virol.

[47] Huang KA, Huang PN, Huang YC, Yang SL, Tsao KC, Chiu CH, Shih SR, Lin TY (2020). Emergence of genotype C1 Enterovirus A71 and its link with an antigenic variation of virus in Taiwan. PLoS Pathog.

[48] Zhang L, Wei M, Jin P, Li J, Zhu F (2021). An evaluation of a test-negative design for EV-71 vaccine from a randomized controlled trial. Hum Vaccines Immunother.

[49] Duan Y, Meng Y, Gao Z, Wang X, Zhang H (2021). microRNA-9-5p protects liver sinusoidal endothelial cell against oxygen glucose deprivation/reperfusion injury. Open Life Sciences.

[50] Marquez YMR, Cid LED, Pertuz LD, Villavicencio MN, Negrón ME (2019). Políticas públicas para una coeducación con equidad. Opc Revista de Cienc Hum y Soc.

[51] Xing J, Liu D, Shen S, Su Z, Zhang L, Duan Y, Tong F, Liang Y, Wang H, Deng F, Hu Z, Zhou Y (2016). Pathologic studies of fatal encephalomyelitis in children caused by enterovirus 71. Am J Clin Pathol.

[52] Ong KC, Badmanathan M, Devi S, Leong KL, Cardosa MJ, Wong KT (2008). Pathologic characterization of a murine model of human Enterovirus 71 encephalomyelitides. J Neuropathol Exp Neurol.

[53] Wong KT, Munisamy B, Ong KC, Kojima H, Noriyo N, Chua KB, Ong BB, Nagashima K (2008). The distribution of inflammation and virus in human enterovirus 71 encephalomyelitis suggests possible viral spread by neural pathways. J Neuropathol Exp Neurol.

[54] Pérez-Vélez CM, Anderson MS, Robinson CC, McFarland EJ, Nix WA, Pallansch MA, Oberste MS, Glodé MP (2007). Outbreak of neurologic Enterovirus type 71 disease: a diagnostic challenge. Clin Infect Dis.

[55] Li R, Zou Q, Chen L, Zhang H, Wang Y (2011). Molecular analysis of virulent determinants of enterovirus 71. PLoS ONE.

[56] Huang HI, Weng KF, Shih SR (2012). Viral and host factors that contribute to the pathogenicity of enterovirus 71. Future Microbiol.

[57] Wang SM, Lei HY, Huang KJ, Wu JM, Wang JR, Yu CK, Su IJ, Liu CC (2003). Pathogenesis of enterovirus 71 brainstem encephalitis in pediatric patients: roles of cytokines and cellular immune activation in patients with pulmonary edema. J Infect Dis.

[58] Gan ZK, Jin H, Li JX, Yao XJ, Zhou Y, Zhang XF, Zhu FC (2015). Disease burden of enterovirus 71 in rural central China: a community-based survey. Hum Vaccin Immunother.

[59] Lin JY, Shih SR (2014). Cell and tissue tropism of Enterovirus 71 and other enteroviruses infections. J Biomed Sci.

[60] Prajapati HJ, Spivey JR, Hanish SI, El-Rayes BF, Kauh JS, Chen Z, Kim HS (2013). mRECIST and EASL responses at early time point by contrast-enhanced dynamic MRI predict survival in patients with unresectable hepatocellular carcinoma (HCC) treated by doxorubicin drug-eluting beads transarterial chemoembolization (DEB-TACE). Ann Oncol.

[61] Tung WS, Bakar SA, Sekawi Z, Rosli R (2007). DNA vaccine constructs against enterovirus 71 elicit an immune response in mice. Genet Vaccines Ther.

[62] Foo DG, Alonso S, Phoon MC, Ramachandran NP, Chow VT, Poh CL (2007). Identification of neutralizing linear epitopes from the VP1 capsid protein of Enterovirus 71 using synthetic peptides. Virus Res.

[63] Chong P, Hsieh SY, Liu CC, Chou AH, Chang JY, Wu SC, Liu SJ, Chow YH, Su IJ, Klein M (2012). Production of EV71 vaccine candidates. Hum Vaccin Immunother.

[64] Zhu FC, Meng FY, Li JX, Li XL, Mao QY, Tao H, Zhang YT, Yao X, Chu K, Chen QH, Hu YM, Wu X, Liu P, Zhu LY, Gao F, Jin H, Chen YJ, Dong YY, Liang YC, Shi NM, Ge HM, Liu L, Chen SG, Ai X, Zhang ZY, Ji YG, Luo FJ, Chen XQ, Zhang Y, Zhu LW, Liang ZL, Shen XL (2013). Efficacy, safety, and immunology of an inactivated alum-adjuvant enterovirus 71 vaccines in children in China: a multicentre, randomized, double-blind, placebo-controlled, phase 3 trial. Lancet.

[65] Reed Z, Cardosa MJ (2016). Status of research and development of vaccines for Enterovirus 71. Vaccine.

